# PAK5 Induces EMT and Promotes Cell Migration and Invasion by Activating the PI3K/AKT Pathway in Ovarian Cancer

**DOI:** 10.1155/2018/8073124

**Published:** 2018-09-02

**Authors:** Diyou Li, Yinglin Pan, Yating Huang, Ping Zhang, Xuhong Fang

**Affiliations:** Department of Obstetrics and Gynecology, Xin Hua Hospital Affiliated to Shanghai Jiao Tong University School of Medicine, 1665 Kongjiang Rd, Shanghai 200092, China

## Abstract

Ovarian cancer is the most lethal gynecologic cancer and currently ranks fifth in causing cancer-related deaths among women. P21^cdc42/rac1^-activated kinase 5 (PAK5) is a newly identified protein that has been indicated to have oncogenic potential. The present study investigated the expression level of PAK5 in clinical ovarian cancer and the functional roles of PAK5 in ovarian cancer progression. It was initially found that PAK5 was highly expressed in ovarian cancer tissues, particularly in patients with distant metastasis. Higher expression of PAK5 predicted poor survival fates in patients with ovarian cancer (*p* = 0.008). Knockdown of PAK5 in SKOV3 cells caused epithelial cell phenotypes, whereas overexpression of PAK5 led to remarkable mesenchymal cell phenotypes in A2780 cells. When PAK5 was depleted from SKOV3 cells, cells exhibited impaired wound recovery abilities. Cell migration and invasion abilities were also significantly inhibited. On the contrary, when PAK5 was overexpressed in A2780 cells, the wound recovery ability was enhanced by 68%. Cell migration and invasion abilities were consistently increased to approximately 2-fold. After knockdown of PAK5, the phosphorylation levels of PI3K p85 at Tyr458 and its downstream AKT at Ser473 were both decreased. The total protein of PI3K and AKT as well as the phosphorylation level of AKT at Thr308 remained unaffected. These data suggested that PI3K induced epithelial-to-mesenchymal transition and promoted cell migration and invasion by activating the PI3K/AKT pathway in ovarian cancer. The oncogenic potential of PAK5 in ovarian cancer might suggest that any therapeutic strategies targeting PAK5 had the promising value for ovarian cancer treatment.

## 1. Introduction

Ovarian cancer is one of the leading causes of cancer-related deaths in females and currently ranks fifth in causing cancer-related deaths among women. Based on a recent statistic, there are 22,280 newly diagnosed cases of ovarian cancer in the United States each year, among which 15,500 are estimated to die each year [1]. Current therapeutic options for ovarian cancer patients consist of surgery, radiotherapy, and chemotherapy. However, most patients relapse after surgery or develop resistance to chemotherapy drugs [2, 3]. Due to untimely diagnosis and limited therapeutic strategies, the prognosis of ovarian cancer patients still remains poor with over 70% estimated patients diagnosed at an advanced stage [2, 3], and the 5-year survival rate is only approximately 30% [4]. Therefore, it is mandatory to find novel targets for the early diagnosis and treatment of ovarian cancer.

P21^cdc42/rac1^-activated kinase 5 (PAK5) was first cloned and characterized in 2002 as a brain-specific kinase and contributed to the formation of filopodia in nerve cells [4]. The gene *pak5* is located in the *Homo sapiens* 20p12 chromosomal locus and encoded by 12 exons and encodes an 80 kDa protein. PAK5 is one of the members of the PAK II subfamily of PAKs and localizes on the mitochondria and the nucleus. In mammals, PAKs (PAK1–6) have been divided into group I (PAK1, PAK2, and PAK3) and group II (PAK4, PAK5, and PAK6) based upon their structure and sequence homology [5]. PAK5 contains 719 amino acid residues and completely discriminates in sequence from other PAKs. PAK5 contains a highly conserved p21-GTPase-binding domain (PBD), which is characteristic in the whole PAK family, so as to interact with GTP-binding Cdc42 preferentially [5].

PAK5 is the last identified and the least understood member of the PAK family [6, 7]. Ever since its identification in brain neuronal cells, mounting evidence has regarded PAK5 as an important mediator of tumor progression. PAK5 has currently been shown to be involved in the regulation of cytoskeleton changes, antiapoptosis, and proliferation in tumor cells [6]. The antiapoptotic properties of PAK5 depend on its nucleocytoplasmic shuttling [8]. The regulation of cytoskeleton-mediating changes by PAK5 has indicated the role of PAK5 in cell morphology, adhesion, and motility. For instance, knockdown of PAK5 in human glioma cells inhibited cell migration and invasion by interacting with EGR1-MMP2 signaling [9]. PAK5-mediated phosphorylation and nuclear translocation of NF-*κ*B-p65 promote breast cancer cell proliferation *in vitro* and *in vivo* [10]. Of great interest, evidence also showed that PAK5 promotes epithelial-to-mesenchymal transition in several types of cancer such as colon cancer [11] and bladder cancer [12]. All these pioneering studies imply that PAK5 has a great potential to mediate cancer progression.

Recently, it has been reported that PAK5 overexpression promotes paclitaxel chemoresistance of epithelial ovarian cancer [13], suggesting that PAK5 may also have a role in the regulation of ovarian cancer. However, no related study has ever been conducted. The present study was aimed at investigating the expression profile of PAK5 in clinical ovarian cancer and at examining its functional roles in ovarian cancer cell epithelial-to-mesenchymal transition and migration and invasion. The underlying mechanisms that contributed to PAK5-mediated biological activities were also assessed in the present study. Our loss-of-function and gain-of-function studies indicated that PAK5 induced epithelial-to-mesenchymal transition and promoted cell migration and invasion by activating the PI3K/AKT pathway in ovarian cancer.

## 2. Materials and Methods

### 2.1. Human Specimen and Ethical Statements

A total of 66 clinical cases that were surgically dissected and diagnosed with ovarian cancer were collected. The paired noncancerous tissues were also obtained from each patient. We also obtained both the primary tumor tissues and the distant metastatic loci from 6 cases with ovarian cancer. In a second follow-up study, the patients with ovarian cancer in our hospital were consecutively followed for 80 months, and the survival rate was calculated based on the expression level of PAK5. All patients showed their full consent to participate in our study, and a written consent form was obtained from each patient. Protocols for using human tissues were approved by the ethical committee board at Xin Hua Hospital Affiliated to Shanghai Jiao Tong University School of Medicine.

### 2.2. Immunohistochemistry (IHC) Analysis

Tissue slides were dewaxed at 60°C for 4 h followed by three 10-min washes with xylene and then rehydrated with gradient ethanol including 100%, 95%, 85%, 75%, and 50% in sequence and finally with distilled water for 5 min each. To retrieve antigen, the slides were put into 10 mM citrate buffer (pH 6.0) when the buffer was heated to 95°C and were kept for 30 min. Then, the endogenous peroxidase activity was inhibited by 3% hydrogen peroxide for 30 min at room temperature. After blocking with 5% normal goat serum for 30 min, the slides were incubated with an anti-PAK5 antibody overnight at 4°C. The sections were then incubated for 1 h with a HRP-conjugated secondary antibody. Immunoreaction was developed using 3,3′-diaminobenzidine (DAB; Zhongshan Biotech, Beijing, China) substrate. After hematoxylin counterstain and dehydration, the sections were sealed with cover slips.

To evaluate the immunostaining, the immunoreactivity was assessed by two independent pathologists in a blinded manner under light microscopy (Olympus BX-51 light microscope), and the image was collected by a Camedia Master C-3040 digital camera. Positive PAK5 immunostaining is defined as cytoplasmic with or without nuclear staining and graded according to both the intensity and percentage of cells with positive staining. Scoring of the PAK5 staining intensity was on a 0–3 scale (0 = negative, 1 = weak, 2 = moderate, and 3 = strong). The percentage of PAK5-positive stained cells was also scored into four categories: 1 (0–25%), 2 (26–50%), 3 (51–75%), and 4 (76–100%). The level of PAK5 staining was evaluated by the immunoreactive score (IRS), which is calculated by multiplying the scores of staining intensity and the percentage of positive cells.

### 2.3. Cells and Reagents

Human ovarian cancer cell lines SKOV3, A2780, OVCAR, HRA, and COC1 were purchased from the Cell Bank of the Chinese Academy of Sciences (Shanghai, China). All cell lines were maintained in Dulbecco's modified Eagle medium (DMEM) (Gibco, Los Angeles, CA, USA) supplied with 10% fetal bovine serum (FBS) (Gibco). Culture medium was refreshed every two days unless otherwise stated. Primary antibodies were commercially purchased from Santa Cruz Biotechnology (Santa Cruz, CA, USA) except for the phosphorylation detection antibodies which were obtained from Cell Signaling Technology (Boston, MA, USA). For knockdown of PAK5, two specific shRNAs were chemically synthesized by GenePharma (Shanghai, China). A scramble negative control shRNA (shNC) was also synthesized serving as control shRNA. A PAK5 expression plasmid was purchased from Addgene Inc. (Cambridge, MA, USA).

### 2.4. Quantitative Real-Time Polymerase Chain Reaction (qRT-PCR)

Total RNAs from both human tissues and cultured cells were extracted with TRIZOL reagent (TaKaRa, Dalian, China). Thereafter, RNAs were reversely transcribed into cDNA using a reverse transcription kit (TaKaRa, Japan). RT-PCR was performed with the SYBR green reagent in an ABI 7900 machine. The primers used in this study were as follows: *pak5* (forward 5′-CCAAAGCCTATGGTGGACCC-3′ and reverse 5′-AGGCCGTTGATGGAGGTTTC-3′) and *gapdh* (forward 5′-GTGGACATCCGCAAAGAC-3′ and reverse 5′-AAAGGGTGTAACGCAACTA-3′).

The relative expression of human PAK5 was normalized to *gapdh* and calculated with the 2^−ΔΔCt^ method.

### 2.5. Western Blot

Cell extracts were obtained in lysis buffer (50 mM Tris (pH 8), 100 mM NaCl, 1% NP-40, 0.5% sodium deoxycholate, 0.5 mM phenylmethylsulfonyl fluoride, and 1 *μ*g/ml aprotinin). Protein concentrations were assessed using a bicinchoninic acid kit (Pierce, CA, USA), and equal amounts of proteins (60 *μ*g) were separated by 10% SDS-polyacrylamide gel electrophoresis (SDS-PAGE) and transferred to polyvinylidene difluoride membranes (Millipore, CA, USA). Blots were blocked for 1 h with Tris-buffered saline containing 0.1% Tween-20 supplemented with 5% nonfat milk and were then incubated with primary antibodies overnight at 4°C. After washing, blots were incubated with HRP-conjugated secondary antibodies, which were detected using the enhanced electrochemical luminescence (ECL) reagent (Fisher Scientific, CA, USA).

### 2.6. Immunofluorescence and Confocal Microscopy

Briefly, SKOV3 and A2780 cells were pretreated as indicated and were cultured on glass cover slips in a 24-well plate. Cells were fixed in 4% paraformaldehyde for 10 min at room temperature and then rinsed 3 times with cold PBS 5 min/time. After being permeabilized with 0.3% Triton-100 for 15 min, cells were blocked with PBS containing 5% bull serum albumin (BSA, Sigma) for 60 min. Subsequently, cells were incubated with the primary antibody against F-actin diluted in blocking buffer overnight at 4°C, followed by visualization with a red-expressing secondary antibody (60 min) diluted at 1 : 1000 in blocking buffer. Nuclei were counterstained with 4′,6-diamidino-2-phenylindole (DAPI) for 10 min. Images were examined and recorded using immunofluorescence confocal laser scanning microscopy (Zeiss LSM 880, Germany).

### 2.7. Wound Healing Assay

SKOV3 and A2780 that were pretreated as indicated were placed on 6-well plates to form a confluent monolayer. Then, artificial wounds were made vertically to each group of cells with sterile pipette tips. Wound recovery was observed every 6 h. The rate of wound recovery (termed as relative migration rate (%)) was then calculated after 18 h monitoring.

### 2.8. Transwell Migration and Invasion Assays

SKOV3 and A2780 that were pretreated as indicated were then trypsinized and suspended in FBS-free medium, and 100 *μ*l of cell suspension was added into the upper chamber of transwell. DMEM supplemented with 5% FBS was placed in the lower chamber. Invasion assay was basically similar to migration assay protocols except that a Matrigel (BD Biosciences) was spread evenly on the microfilm of a transwell chamber prior to the assay. After 24 h of free transmigration, transmembrane cells were washed and stained with crystal violet. The cells in five randomly selected visions of each group were counted under an inverted microscope (Nikon, Japan).

### 2.9. Statistical Analysis

All data were presented as mean ± standard deviation (SD). Student's *t*-test was used to compare the difference between the control and the experimental group. The Kaplan-Meier method was performed to assess the survival rate difference by means of the log-rank (Mantel-Cox) test. Any value of *p* < 0.05 was considered a significant difference. Each experiment was repeated in triplicate.

## 3. Results

### 3.1. PAK5 Was Highly Expressed in Ovarian Cancer and Predicted Poor Prognosis

**Figure 1:**
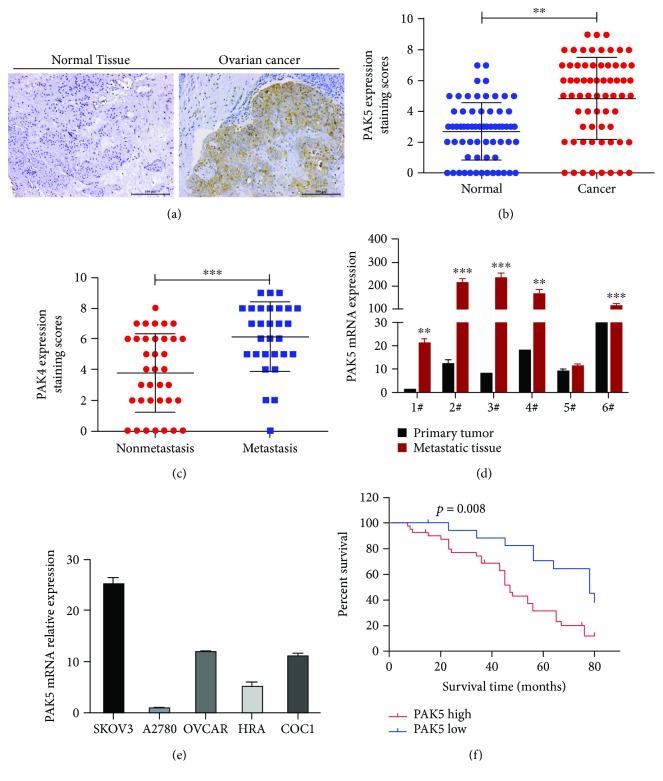
PAK5 was highly expressed in ovarian cancer and predicted poor prognosis. (a) IHC staining of PAK5 in clinical ovarian cancer and paired noncancerous tissues (*n* = 66 for each group). Scale bar = 100 *μ*m. (b) The IHC staining results were scored based on staining intensity (0–3 score) and percentage of positive cells (1–4 score). Immunoreactive score (IRS) was then calculated by multiplying intensity score and percentage of positive cells and represented the PAK5 staining score. (c) The 66 ovarian cancer cases were divided into metastasis and nonmetastasis cases. The IHC staining of these metastasis or nonmetastasis cases were scored, and the IRS staining score was shown. (d) Six cases that had distant metastasis were selected for qRT-PCR. The mRNA level of PAK5 in metastatic loci and in the primary tumor was compared. (e) The mRNA levels of PAK5 in a series of ovarian cancer cell lines were detected using qRT-PCR analysis. (f) Survival rate analysis of ovarian cancer patients that had either high or low level of PAK5. ^∗∗^*p* < 0.01 and ^∗∗∗^*p* < 0.0001.

Initially, we collected 66 cases of clinical ovarian cancer as well as paired normal noncancerous tissues. Expression of PAK5 in clinical tissues was assessed using IHC analysis, and the staining intensity was scored on a 0-to-7 scale. IHC analysis showed that PAK5 was significantly stained in ovarian cancer tissues, while it was barely detected in normal tissue (Figure 1(a)). The score of the immunoreactivity showed that the mean IRS score of PAK5 staining was significantly elevated in cancerous tissues, making an approximately 2-fold increase of that in normal tissues (Figure 1(b)). After dividing all the 66 ovarian cancer cases into metastasis and nonmetastasis cases, it was further found that the IRS staining score in metastasis cases was significantly higher than that in nonmetastasis cases (Figure 1(c)). In 6 cases, qRT-PCR analysis showed that 5 cases showed significantly higher mRNA of PAK5 in the metastatic tissues than in the primary loci (Figure 1(d)). qRT-PCR analysis also showed that the mRNA level of PAK5 was differentially expressed in a series of ovarian cancer cell lines, including SKOV3, A2780, OVCR, HRA, and COC1, whereas the highest level presented in SKOV3 cells and the least level in A2780 cells. More importantly, in an 80-month follow-up study, it was found that the patients with a higher PAK5 expression had significantly lower survival rate. Only less than 20% of patients with higher PAK5 levels survived, which contradicted dramatically to more than 40% survival rate in patients with lower PAK5 levels (Figure 1(f)). These clinical observations suggested that PAK5 was highly expressed in ovarian cancer and predicted poor prognosis.

### 3.2. Knockdown of PAK5 Caused the Mesenchymal-to-Epithelial Transition in SKOV3 Cells

**Figure 2:**
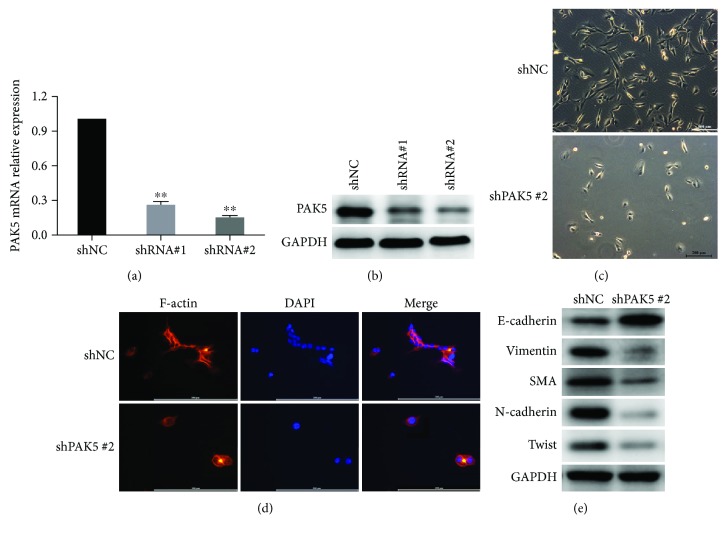
Knockdown of PAK5 caused the mesenchymal-to-epithelial transition. (a, b) qRT-PCR and western blot analysis of PAK5 levels in SKOV3 cells with a scramble negative control shRNA (shNC) or two specific shRNAs against PAK5 (denoted as shRNA#1 and shRNA2). Morphology of SKOV3 cells after transfection with shNC or the second shRNA against PAK5 (shRNA#2). (d) Immunofluorescence analysis of F-actin, one of the filament units indicating cell motility, in SKOV3 cells with shNC or shRNA#2. DAPI was counterstained to manifest cell nuclei. Merged images were also shown. (e) Western blot analysis of epithelial marker E-cadherin and mesenchymal markers N-cadherin, Twist, vimentin, and smooth muscle actin (SMA) in control of PAK5-depleted SKOV3 cells. ^∗∗^*p* < 0.01 versus shNC.

In view of the fact that SKOV3 had the highest level of PAK5 and A2780 had the least level of PAK5, we then synthesized two specific shRNAs against PAK5 (denoted as shRNA#1 and shRNA#2, resp.) to knock down its expression in SKOV3 cells. qRT-PCR analysis showed that either shRNA significantly depletes the mRNA level of PAK5, while the second shRNA (shRNA#2) had better knockdown efficiency (Figure 2(a)) and shRNA#2 was hence adopted for subsequent analyses. Western blot analysis also confirmed that either shRNA transfection caused a marked decrease in PAK5 protein level (Figure 2(b)). Interestingly, it was observed that after knockdown of PAK5 in SKOV3 cells, cells adopted an apico-basal polarized morphology (Figure 2(b)). Immunofluorescence analysis also showed that knockdown of PAK5 caused decreased expression of F-actin, an indicator for cell motility (Figure 2(d)). Furthermore, Western blot analysis showed that the protein level of epithelial marker E-cadherin was significantly elevated by knockdown of PAK5 using the shRNA#2. On the contrary, mesenchymal markers, including vimentin, smooth muscle actin (SMA), N-cadherin, and Twist, were consistently decreased by knockdown of PAK5 in SKOV3 cells (Figure 2(e)). All these data suggested that knockdown of PAK5 decreased cell motility and led to cell morphology changes into a mesenchymal phenotype.

### 3.3. Reexpression of PAK5 in A2780 Cells Promoted Epithelial-to-Mesenchymal Transition

**Figure 3:**
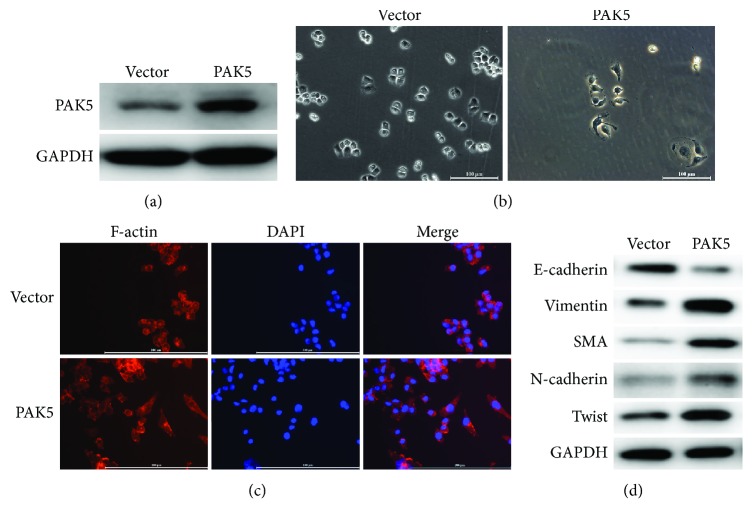
Reexpression of PAK5 in A2780 cells promoted epithelial-to-mesenchymal transition. (a) A2780 cells that had the least expression of PAK5 were transfected with an expression plasmid of PAK5 to reexpress it. An empty vector was also transfected into A2780 to serve as control. Western blot analysis of PAK5 expression was performed to confirm the working efficiency of PAK5 plasmid. (b) Cell morphology was shown in control (vector-transfected) and PAK5-overexpressed A2780 cells. (c) Immunofluorescence assay was performed to shown F-actin expression in A2780 cells. (d) Western blot analysis of epithelial marker E-cadherin and major mesenchymal markers such as vimentin, SMA, N-cadherin, and Twist in control and PAK5-overexpressed A2780 cells.

Subsequently, A2780 cells that had the least expression of PAK5 were transfected with an expression plasmid of PAK5 to reexpress it in A2780 cells. The expression plasmid remarkably increased the protein level of PAK5 in A2780 cells (Figure 3(a)), confirming the working efficiency of PAK5 plasmid. In contrast to the control A2780 cells, PAK5-overexpressed A2780 cells exhibited an apico-basal polarization in morphology, an indication of a mesenchymal cell phenotype (Figure 3(b)). F-actin was highly expressed in A2780 cells after reexpression of PAK5, as evidenced by the immunofluorescence analysis (Figure 3(c)). Consistent with the morphology observation, it was found that reexpression of PAK5 led to decreased expression of E-cadherin, whereas it increased the protein levels of mesenchymal markers including vimentin, SMA, N-cadherin, and Twist (Figure 3(d)). All these data suggested that the overexpression of PAK5 promoted epithelial-to-mesenchymal transition in A2780 cells.

### 3.4. Knockdown of PAK5 Inhibited Cell Migration and Invasion Abilities in SKOV3 Cells

**Figure 4:**
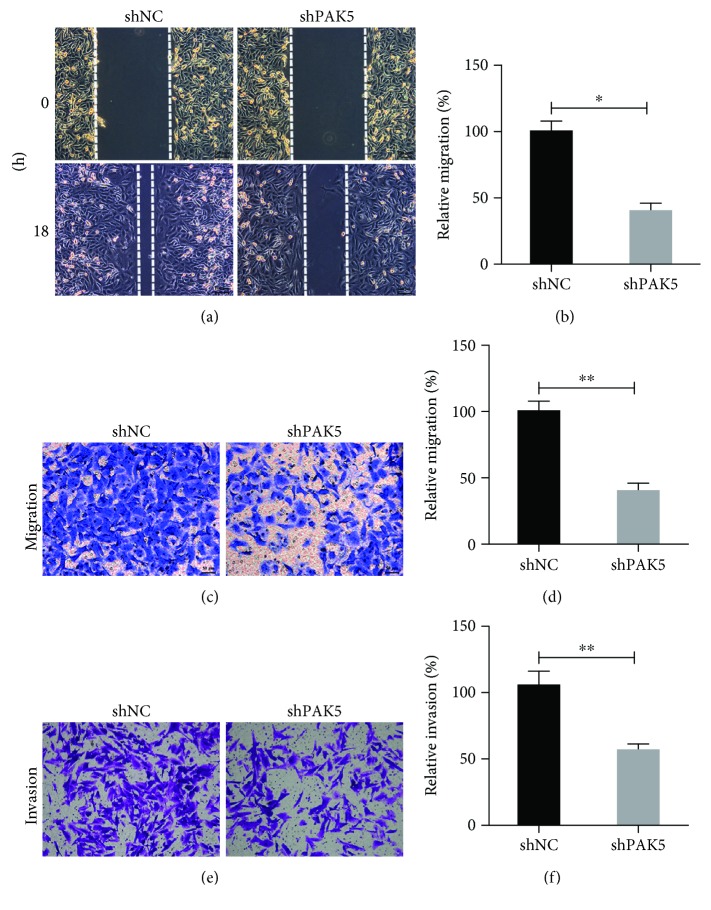
Knockdown of PAK5 inhibited cell migration and invasion abilities in SKOV3 cells. (a, b) Wound healing assay was performed in shNC and shPAK5 transfected SKOV3 cells. Cells were initially transfected with respective shRNA and wounds were shown 18 h after scratch. And the relative migration (%), which was determined by the percentage of wound recovery area, was quantitatively analyzed for each group. (c, d) Cell migration assay was performed and the transmigrated cells were stained with crystal violet. Transmigrated cells were also manually counted for each group. (e, f) Cell invasion assay was performed and the invaded cells were stained with crystal violet. Invaded cells were also manually counted for each group. ^∗^*p* < 0.05; ^∗∗^*p* < 0.01.

In the wound healing assay, it was observed that control SKOV3 cells showed an active recovery ability of scratched wound. However, in the SKOV3 cells that were depleted of PAK5, the marked wound was observed 18 h after shRNA#2 (shPAK5) transfection (Figure 4(a)). In fact, the wounds were only approximately 40% recovered after 18 h of shPAK5 transfection, which was significantly lower than that of the control SKOV3 cells (Figure 4(b)). The cell migratory ability was also reflected by the cell migration assay, where there were visibly less SKOV3 cells that transmigrated into the lower surface of the membrane after PAK5 depletion (Figure 4(c)). Counting of the migrated cells showed that there was only an average of 36 SKOV3 cells transmigrated in the PAK5-depleted group, which remarkably contradicted to almost 100 cells in the control SKOV3 cells (Figure 4(d)). SKOV3 cells also exhibited decreased invasive abilities after knockdown of PAK5 as shown by the invasion assay after crystal violet staining (Figure 4(e)). Quantitation of invaded cells showed that the invasive ability of PAK5-depleted SKOV3 cells was only half of the control SKOV3 cells (Figure 4(f)). All these data suggested that knockdown of PAK5 inhibited cell migration and invasion in SKOV3 cells.

### 3.5. Overexpression of PAK5 Promoted Cell Migration and Invasion in A2780 Cells

**Figure 5:**
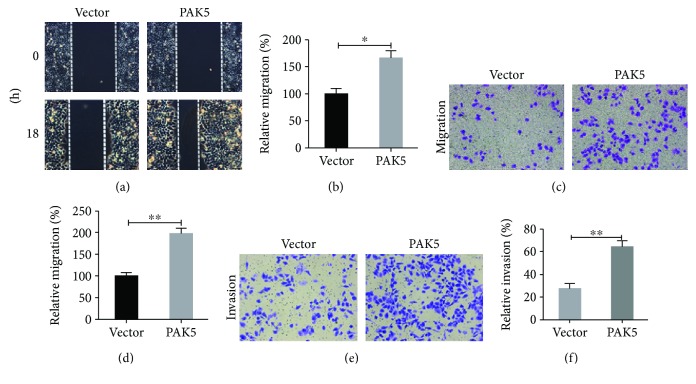
Overexpression of PAK5 promoted cell migration and invasion in A2780 cells. (a, b) Wound healing assay was performed in vector and PAK5 plasmid- transfected A2780 cells. Wounds were shown 18 h after scratch. And the relative migration (%), determined by the percentage of wound recovery area, was quantitatively analyzed for each group. (c, d) Cell migration assay was performed and the transmigrated cells were stained with crystal violet. Transmigrated cells were manually counted for each group. (e, f) Cell invasion assay was performed and the invaded cells were stained with crystal violet. Invaded cells were also manually counted for each group. ^∗^*p* < 0.05; ^∗∗^*p* < 0.01.

On the other way, A2780 cells with PAK5 overexpression were subjected to cell migration and invasion assessment. In the wound healing assay, it was found that A2780 cells showed a relatively slow recovery ability of the scratched wounds within 18 h. However, the A2780 cells with PAK5 overexpression actively recovered the wounds, enhancing the wound recovery rate by nearly 68% as compared with control cells (Figures 5(a) and 5(b)). In the cell migration assay, more A2780 cells with PAK5 overexpression were stained underneath the membrane surface (Figure 5(c)). PAK5-overexpressed A2780 cells showed an approximately 2-fold increase in migration ability (Figure 5(d)). Similarly, in the cell invasion assay, more cells were stained in the bottom surface after PAK5 overexpression in A2780 cells (Figure 5(e)). Quantitation of invaded cells showed that PAK5 overexpression led to a 121% increase in cell invasive abilities (Figure 5(f)). These observations suggested that overexpression of PAK5 promoted cell migration and invasion in A2780 cells.

### 3.6. Knockdown of PAK5 Inactivated the PI3K/AKT Pathway

**Figure 6:**
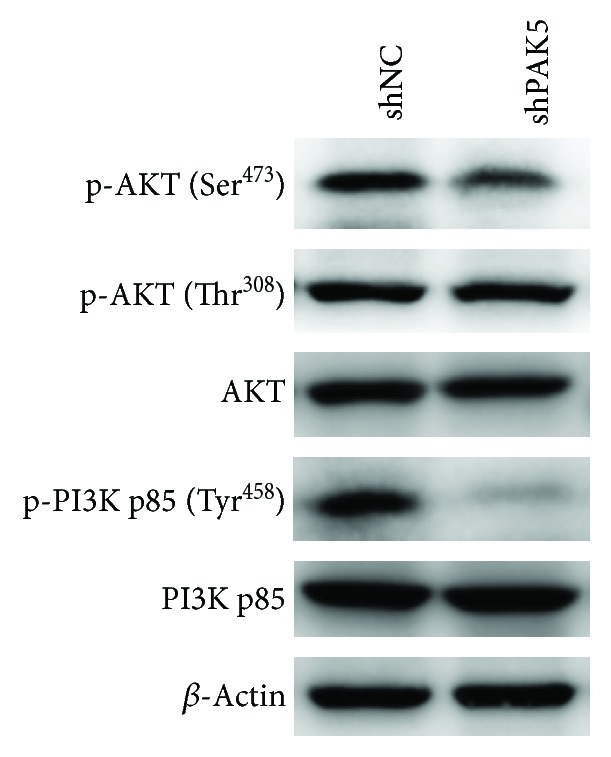
Knockdown of PAK5 inactivated the PI3K/AKT pathway. The phosphorylation levels of PI3K p83 at Tyr458 (p-PI3K p85 at Tyr458) and phosphorylation levels of AKT at Ser473 and Thr308 were detected. The total protein levels of AKT and PI3K p85 were also determined.

Mechanistically, it was detected that after knockdown of PAK5 in SKOV3 cells, the phosphorylated levels of PI3K p85 (Tyr458) were decreased. Accordingly, the downstream AKT at its phosphorylation level at Ser473 was also inhibited by PAK5 depletion. The phosphor-AKT at Thr308 was not altered by PAK5 depletion. Unchanged levels were also observed for the total protein of PI3K p85 and AKT. These data suggested that knockdown of PAK5 inactivated the PI3K/AKT pathway (shown in Figure 6).

## 4. Discussion

Ovarian cancer is one of the most lethal gynecologic cancers because it is most often diagnosed at an advanced stage (60–74%) [14]. As one primary cause of cancer-related deaths for women worldwide, the prognosis of patients with ovarian cancer remains poor due to untimely diagnosis and limited therapeutic strategies. Development of novel therapeutic or diagnostic strategies is therefore urgent for ovarian cancer. To date, it is well documented that a common feature of ovarian cancer is an uncontrolled cell growth and metastasis mechanism [14]. Thus, identification of novel molecules associated with ovarian cancer cell migration and invasion may aid in developing more effective therapies.

In the present study, we identified PAK5 as a critical mediator of ovarian cancer progression. PAK5 was initially found to be overexpressed in clinical ovarian cancer, and PAK5 expression is associated with distant metastasis and poor prognosis. The high expression of PAK5 in ovarian cancer was basically in accordance with that in glioma [9] and in colorectal carcinoma [15].

More importantly, it was found that knockdown of PAK5 in SKOV3 cells led to cell morphology changes and the decreased expression of cytoskeleton F-actin, whereas overexpression of PAK5 in A2780 cells adopted an apico-basal polarization. This observation was consistent with previous literature that regarded the regulations of cytoskeleton-mediating changes in cell morphology, adhesion, and motility as the well-characterized function in tumor progression of PAK5 [6]. In view of the fact that morphological change of the cytoskeleton greatly impacts cancer cell motility, penetration, and angiogenesis and thus results in the occurrence of nearby invasion and distant metastasis [16], the effects of PAK5 on cell migration and invasion were then examined. It was observed that after depletion of PAK5, SKOV3 cells presented with epithelial cell phenotypes and overexpression of PAK5 led to remarkable mesenchymal cell phenotypes in A2780 cells. The morphological alterations that cells undergo during epithelial-to-mesenchymal transition are accompanied by changes in the expression of a large number of molecules [17]. In accordance, the epithelial marker E-cadherin was promoted, while mesenchymal markers vimentin, N-cadherin, and Twist were inhibited after PAK5 depletion. All these observations suggested that PAK5 promoted the epithelial-to-mesenchymal transition in ovarian cancer cell lines. Moreover, when PAK5 was depleted from SKOV3 cells, cells exhibited impaired wound recovery abilities. Cell migration and invasion abilities were also significantly inhibited. On the contrary, when PAK5 was overexpressed in A2780 cells, the wound recovery ability was enhanced by 68%. Cell migration and invasion abilities were consistently increased to approximately 2-fold. Our observations suggested that PAK5 induced epithelial-to-mesenchymal transition and promoted cell migration and invasion in ovarian cancer. Considering that PAK5 also regulated cell migration and invasion in cancer types such as colon cancer [11, 15], bladder cancer [12], and breast cancer [18], other publication and our findings might implicate that PAK5 had a wide role in regulating solid tumor progression.

The PI3-kinase/AKT pathway (PI3K/AKT) integrates signals from external cellular stimuli to regulate essential cellular functions and is frequently aberrantly activated in human cancers [19]. PI3Ks function as heterodimers that consist of a regulatory p85 subunit and a catalytic p110 subunit. In response to exogenous stimulation, G protein-coupled receptors (GPCRs) or receptor tyrosine kinases (RTKs) are activated, and PI3Ks are recruited to the cellular membrane. The membrane PI3K is activated through phosphorylation at Tyr458, and the activated PI3K subsequently catalyzes the phosphorylation of PtdIns 4,5-bisphoshate (PIP2) to generate the lipid second messenger PtdIns 3,4,5-triphosphosate (PIP3) [4]. As such, the PIP3 second messenger activates downstream signaling pathways, many of which diverge downstream of AKT [19]. AKT is a central conduit of PI3K signaling, and PI3K modulates essential cellular functions utilizing AKT-dependent mechanisms [20]. Upon activation by phosphorylation, AKT modulates various cellular functions including cell survival, proliferation, growth, migration, and invasion through the phosphorylation of more than 200 identified substrates [19]. In the present study, we found that after knockdown of PAK5 in SKOV3 cells, the phosphorylation levels of PI3K (at Tyr458) and AKT (at Ser473) were accordingly decreased, while their total protein levels remained unchanged. Our data suggested that PAK5 regulated the activation process of the PI3K/AKT pathway; in other words, PI3K/AKT signaling might underlie the molecular mechanism that contributed to PAK5-mediated ovarian cancer progression.

In all, the present study identified PAK5 as a critical mediator of ovarian cancer cell migration and invasion. PAK5 also induced epithelial-to-mesenchymal transition in ovarian cancer cells. PAK5 conferred an oncogenic property mainly through regulating the PI3K/AKT pathway. Our data suggested that any therapeutic strategies against PAK5 might serve as a promising avenue to the treatment of ovarian cancer. Small inhibitors targeting the PI3K/AKT activation might also blunt PAK5-mediated biological activities and open novel insights into ovarian cancer treatment.

## Data Availability

The data used to support the findings of this study are available from the corresponding author upon request.
